# Smoking Pack Years and Eustachian Tube Dysfunction

**DOI:** 10.1002/oto2.166

**Published:** 2024-07-05

**Authors:** Arjun Sharma, Sam MacDowell, Nihal Punjabi, Sameer Kejriwal, Vikram Sharma, Jared C. Inman

**Affiliations:** ^1^ School of Medicine California University of Science and Medicine Colton California USA; ^2^ Department of Otolaryngology–Head and Neck Surgery Loma Linda University Health Loma Linda California USA; ^3^ School of Medicine Case Western Reserve University Cleveland Ohio USA; ^4^ John A. Burns School of Medicine University of Hawaii Honolulu Hawaii USA; ^5^ College of Letters and Sciences University of California, Los Angeles Los Angeles California USA

**Keywords:** eustachian tube dysfunction, risk factors, smoking

## Abstract

**Objective:**

To determine the effect of smoking history on the risk of developing obstructive eustachian tube dysfunction (OETD).

**Study Design:**

Cross‐sectional review.

**Setting:**

National database.

**Methods:**

Data from the National Health and Nutrition Examination Survey (1999 to present) was analyzed. OETD was defined as middle ear pressure less than −100 decapascals (daPa). Nonsmokers, current smokers, with tympanometry data were analyzed. Patients under the age of 18, with myringotomy tubes, or with a sinus problem/earache/cold in the past 24 hours were excluded. The relative risks (RRs) for developing OETD were calculated for nonsmokers versus smokers and those with greater versus less than 10, 20, and 30 pack years (py).

**Results:**

A total of 9472 patients met inclusion criteria (54.1% female, 75.9% non‐Hispanic, mean age 43, 20.3% smokers). The RR of having OETD for smokers versus nonsmokers was 1.75 [95% confidence interval, CI: 1.45‐2.11]. The RR of having OETD for patients with a 10+ py was 1.97 [95% CI 1.57‐2.47], 20+ py was 2.29 [95% CI 1.76‐2.95], and 30 py or greater was 2.08 [95% CI 1.49‐2.90].

**Conclusion:**

In this study, smoking roughly doubled the risk of developing OETD, as represented by a single measurement of negative middle ear pressure less than −100 daPa. The definition of OETD used in this study was limited, as it did not include symptomology, and more work is needed to examine additional covariates. However, these results may guide future research to better counsel and screen patients for OETD.

The effect of smoking on eustachian tube dysfunction is well established in anecdotal clinical practice; however, the quantitative relationship between smoking and eustachian tube dysfunction has never been established. Estimated to affect 4.6% of adults aged 20 or older in United States, eustachian tube dysfunction is the failure of the eustachian tube to properly ventilate and balance pressure between the middle ear and nasopharynx.^1^, ^2^ Obstructive eustachian tube dysfunction (OETD), the most common subtype, is thought to occur due to the inability of the structure to effectively dilate.^1^, ^2^ OETD is diagnosed by a combination of symptoms, physical exam findings, and tympanometry.^2^ Past literature and current consensus guidelines consider a type C tympanogram or a peak pressure of less than −100 decapascals (daPa) necessary to diagnose OETD.^2^, ^3^, ^4^, ^5^, ^6^, ^7^

Despite advances in diagnosis and treatment, the pathophysiology of OETD is not fully understood.^1^, ^2^, ^3^, ^8^ Inflammatory conditions, such as respiratory tract infections, contribute significantly to the development of acute OETD, but studies exploring potential risk factors for chronic OETD, such as smoking and body mass index, are sparse.^3^, ^9^, ^10^, ^11^ In the pediatric population, a recent systematic review and meta‐analysis found that over 290,000 cases of childhood middle ear disease each year are attributed to secondhand tobacco smoke exposure.^12^ Furthermore, Patel and colleagues reconciled the results of prior studies, showing that children exposed to high concentrations of environmental tobacco smoke had significantly higher odds of having OETD, defined by middle ear pressure less than −100 daPa.^5^, ^13^ In adults, a similar large retrospective study showed a significant association between tobacco smoke exposure and middle ear diseases. However, links between smoking and objectively measured OETD are limited to small observational and animal studies.^11^, ^14^, ^15^, ^16^ None of these studies have measured the risk of OETD by the dose of smoke exposure like the work that has been done in the chronic obstructive pulmonary disease (COPD) literature.^11^, ^17^, ^18^, ^19^ For instance, a large case‐control study of 57,779 patients found that the odds of contracting COPD almost doubled when a patient reaches 20 pack years (py) or more.^17^

To our knowledge, no study has explored the relationship between active smoking and OETD in a large representative sample of the US adult population. In addition, no other study has attempted to map a possible dose‐dependent pattern between smoking py and the likelihood of a patient having negative middle ear pressure. In this retrospective cross‐sectional study, the National Health and Nutrition Examination Survey (NHANES) database is analyzed to answer these questions.

## Methods

This study was exempt for ethical review by the Loma Linda University Institutional Review Board, as only deidentified information from a publicly available database was analyzed.

### Study Population

NHANES is a multiyear, cross‐sectional epidemiology study that collects symptoms, lifestyle, physical examination, and other evaluations—including tympanometry—data from a representative US sample.^20^ We analyzed all available demographic, audiometric, and smoking questionnaire data from the NHANES (1999 to present). Data from 2021 to 2023 has yet to be made available and could not be a part of this analysis. Data from 2019 to 2020 was not completed due to the COVID‐19 pandemic. Data from 2013 to 2014 was also excluded due to no tympanometry data being collected in that year. Nonsmokers, current smokers, and those with bilateral tympanometry data available were analyzed. py was calculated using the smoking questionnaire from NHANES.

Former smokers were excluded from our primary analysis but included in a separate analysis. Patients under the age of 18 were excluded as this study only focuses on adults. Patients with myringotomy tubes or a sinus problem/earache/cold in the past 24 hours were also excluded because these factors would alter middle ear pressure. While myringotomy tubes may be indicative of OETD, only 34 patients in the entire cohort had them, so no statistical analyses could be based on this characteristic. Finally, patients without tympanometry data available for both ears were also excluded.

### Tympanometry Measurements

Standard tympanometry was carried out during the NHANES survey to collect middle ear pressure data. The primary outcome of OETD was defined as a peak mean middle ear pressure less than –100 daPa.^2^, ^3^, ^4^, ^5^, ^6^, ^7^

### Statistical Analysis

The relative risks (RRs) for developing OETD were calculated for nonsmokers versus smokers and patients with greater versus less than 10, 20, and 30 py. Each RR represents the increased risk of having OETD if patients were above a certain py cutoff compared to patients below this cutoff (ie, the increased risk of patients who have a >/=10 py history compared to those with a <10 py history). Attributable risk and number needed to harm were also estimated from these values. Logistic regression analysis was carried out to assess the influence of other demographic variables on OETD. The same RR analysis was carried out in nonsmokers versus former smokers. All data analysis was carried out using Statistical Package for the Social Sciences Software version 28.0.1.0.

## Results

### Demographics

**Figure 1:**
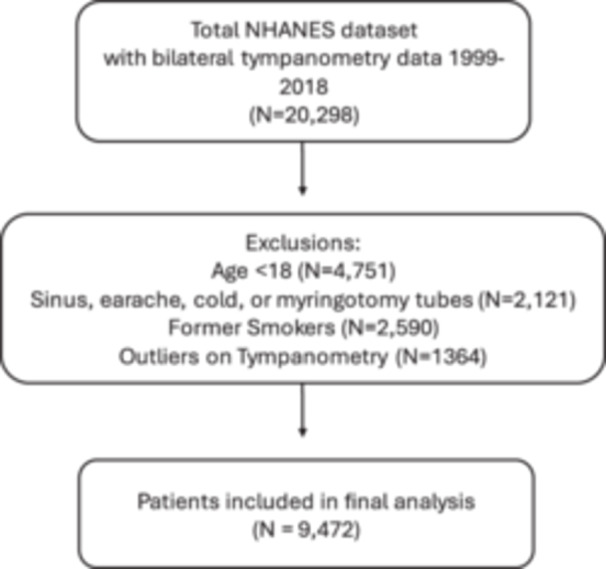
Patient Selection Algorithm using National Health and Nutrition Examination Survey (NHANES) (1999‐2018).

In total, 9472 patients were included (Figure 1). The mean age was 43, 20.3% were smokers, 75.9% were non‐Hispanic, and 54.1% were female. 4.9% of patients met our definition of OETD. Other demographic characteristics are presented in Table 1.

**Table 1:** Demographic Characteristics of Adults Participating in NHANES (1999‐2018) by OETD (n = 9472)

	Eustachian tube dysfunction	
	Present (n = 468)	Absent (n = 9004)
Sex %		
Female	50.0	54.4
Age		
Mean (SD)	49 (19)	42 (18)
Ethnicity %		
Mexican	16.5	18.4
Other Hispanic	9.2	9.3
White	39.3	36.0
Black	25.2	24.0
Other	9.8	12.4
Weight		
BMI > 30	37.6	34.3
BMI < 30	62.4	65.7
Smoker		
% Yes	30.8	19.7

Abbreviation: BMI, body mass index; NHANES, National Health and Nutrition Examination Survey; OETD, obstructive eustachian tube dysfunction; SD, standard deviation.

### RR Analysis

**Figure 2:**
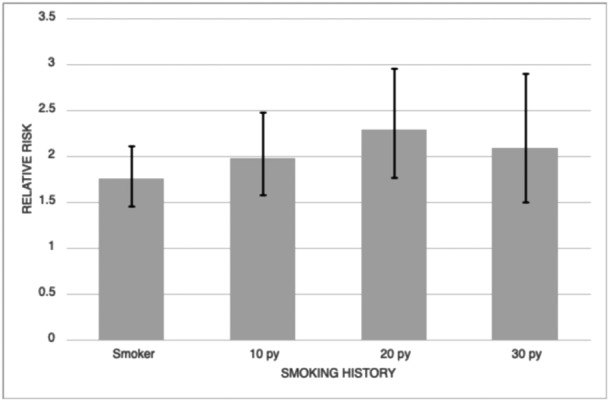
Relative risk of obstructive eustachian tube dysfunction by smoking history. py, pack years

The RR of having OETD was 1.75 [95% confidence interval, CI: 1.45‐2.11] for smokers compared to nonsmokers. The RR of having OETD for patients with a 10+ py was 1.97 [95% CI: 1.57‐2.47], 20+ py was 2.29 [95% CI: 1.76‐2.95], and 30+ py was 2.08 [95% CI: 1.49‐2.90] (Table 2, Figure 2).

**Table 2:** Relative Risk of OETD by Smoking History With Attributable Risk and Number Needed to Harm

Category	Relative risk [95% CI]	Attributable risk (%)	Number needed to harm
Smoker	1.75 [1.45, 2.11]	4.29	23
10 py+	1.97 [1.57, 2.47]	4.92	20
20 py+	2.29 [1.76, 2.95]	5.63	18
30 py+	2.08 [1.49, 2.90]	5.19	19

Abbreviations: CI, confidence interval; OETD, obstructive eustachian tube dysfunction; py, pack years.

### Logistics Regression Analysis

In the logistic regression analysis, the only significant demographic predictor of having OETD from our sample was smoker status. Sex and ethnicity were not seen to have a significant influence (Table 3).

**Table 3:** Logistic Regression Analysis to Assess for Other Predictors of OETD

Variable	OR [95% CI]	*P* value
Smoker	1.74 [1.42, 2.15]	<.001
BMI > 30	1.15 [0.95, 1.40]	.152
Male	0.89 [0.74, 1.08]	.244
Mexican	1.12 [0.77, 1.63]	.550
Other Hispanic	1.21 [0.79, 1.86]	.383
White	1.30 [0.93, 1.81]	.126
Black	1.24 [0.87, 1.76]	.233

Abbreviations: BMI, body mass index; CI, confidence interval; OETD, obstructive eustachian tube dysfunction; OR, odds ratio.

The RR of OETD in smokers compared to nonsmokers was performed in patients under 50 (1.51 95% CI 1.02 to 2.23) and over 50 (1.49, 95% CI: 1.09‐2.04).

### RR of OETD in Former Smoker

In our analysis for former smokers compared to nonsmokers, the RR of having OETD was 1.51 [1.24, 1.83] for former smokers overall, 1.97 [1.60, 2.42] for former 10+ py, 1.94 [1.52, 2.46] for former 20+ py, and 2.02 [1.54, 2.65] for former 30+ py.

## Discussion

This study demonstrates that the risk of experiencing OETD as defined by a single tympanometry value is increased in smokers compared to nonsmokers and doubles in patients with a 10 to 30 py history. Similar results were found for former smokers, suggesting chronic changes despite smoking cessation. Previous animal studies have shown tobacco smoke exposure can decrease the eustachian tube's ability to equalize pressure and cause histologic changes to the mucosa.^15^, ^16^, ^21^, ^22^ Dubin et al demonstrated that 7 to 15 smoke exposures led to negative pressures in the eustachian tubes of rats compared to positive pressure seen with 1 exposure, likely due to acute inflammation and edema.^16^ Histologic changes in the eustachian tube have seen conflicting results, with some describing goblet cell hyperplasia and others describing goblet cell depletion.^15^, ^21^, ^22^ Given the established pathophysiology of COPD, where a reactive goblet cell hypertrophy is seen, the OETD associated with smoking that we describe here may be explained through a similar mechanism.^23^ Altered goblet cells may lead to increased mucus production, effectively clogging the eustachian tube.

Currently, the only other study that directly attempted to explore how smoking impacted eustachian tube function was a small cross‐sectional analysis performed by Pezzoli et al in 2017.^11^ In 64 patients, they found that smokers were significantly more likely to experience OETD, defined as an inability to alter middle ear pressure by 10 mm/H_2_O during swallowing.^11^ This study did not map the risk of OETD by smoke py.

These results also support the previous studies done in the pediatric population looking at secondhand smoke exposure and obstructive OETD.^5^, ^12^, ^13^ Using the same definition of OETD as we did here and NHANES data from 2005 to 2010, Patel et al found that children exposed to higher amounts of environmental tobacco smoke may have a higher prevalence of OETD.^5^ Dating back to 1989, Strachan et al had also described a similar association with one third of middle ear effusions being attributable to passive smoke exposure.^13^

A strength of this study lies in the benefit of a large sample size that is representative of the US population. In addition, NHANES allowed for the control of known confounding variables such as the presence of myringotomy tubes and history of recent upper respiratory tract infections. Since OETD is defined by a combination of symptoms, physical exam findings and tympanometry, the major limitation of this study is only being able to define OETD using the cutoff of –100 daPA.^1^, ^2^, ^8^ Other than trouble hearing, subjective patient‐reported symptoms related to OETD, such as aural fullness, are not reported within NHANES, and hearing difficulty was asked about in different ways from year to year. Consequently, this study could not relate smoking status to OETD symptomology. However, the pressure cutoff of –100 daPA is supported by previous literature and consensus statements that relate this value to OETD symptom scores.^2^, ^4^, ^6^, ^7^ Furthermore, tympanogram data was only available at one point in time, even though true OETD is a chronic process.

Logistic regression analysis suggests that sex and ethnicity do not play a significant role in the risk of OETD in this cohort. The risk effect of smoking on OETD held true in patients under 50 as well as over 50, suggesting that smoking influences risk of OETD independent of age group.

Although this analysis attempted to account for all confounding variables found within literature—namely recent history of upper respiratory tract infection, children, and the presence of myringotomy tubes—it is probable that further analysis might reveal other covariate effects. Patients with symptoms of upper respiratory tract infection reported within 24 hours were excluded, but this timeframe likely does not account for the lingering effects of infection on middle ear pressure even after symptoms have resolved. Future work should further control for this confounder. Additional covariates might include alcohol, other recreational drug use, and age. In addition, it is currently not known whether increasing the length of time from smoking cessation improves eustachian tube function. By comparison, it has been widely shown that smoking cessation delays the advancement of COPD in chronic smokers and has an improved disease course.^24^, ^25^

This work may be used to identify patients who are at higher risk for OETD. Furthermore, by understanding the dose‐dependent relationship between py and risk for OETD, providers may better counsel smokers to cut back or quit in order to optimize ear health.

## Author Contributions

**Arjun Sharma**, conceptualization, data curation, formal analysis, investigation, methodology, project administration, resources, software, validation, visualization, writing—original draft preparation, writing—review and editing; **Sam MacDowell**, conceptualization, data curation, formal analysis, investigation, methodology, software, validation, visualization, writing—original draft preparation, writing—review and editing; **Nihal Punjabi**, conceptualization, investigation, methodology, project administration, visualization, writing—review and editing; **Sameer Kejriwal**, conceptualization, investigation, methodology, writing—original draft preparation, writing—review and editing; **Vikram Sharma**, conceptualization, investigation, methodology, writing—original draft preparation; **Jared C. Inman**, conceptualization, formal analysis, funding acquisition, investigation, methodology, project administration, resources, supervision, validation, visualization, writing—review and editing.

## Disclosures

### Competing interests

The authors declare that there is no conflict of interest.

### Funding source

This research received no specific grant from any funding agency in the public, commercial, or not‐for‐profit sectors.

## Data Availability

The database analyzed in this study is publicly available and accessible at https://www.cdc.gov/nchs/nhanes/index.htm.
